# Antibiotic Prescribing and Doctor-Patient Communication During Consultations for Respiratory Tract Infections: A Video Observation Study in Out-of-Hours Primary Care

**DOI:** 10.3389/fmed.2021.735276

**Published:** 2021-12-01

**Authors:** Annelies Colliers, Katrien Bombeke, Hilde Philips, Roy Remmen, Samuel Coenen, Sibyl Anthierens

**Affiliations:** ^1^Department of Family Medicine and Population Health (FAMPOP), University of Antwerp, Antwerp, Belgium; ^2^Skills Laboratory, University of Antwerp, Antwerp, Belgium; ^3^Vaccine and Infectious Disease Institute (VAXINFECTIO)—Laboratory of Medical Microbiology, University of Antwerp, Antwerp, Belgium

**Keywords:** antibiotics, respiratory tract infections, video observation, reason for encounter, communication, primary care, general practice, out-of-hours care

## Abstract

**Objective:** Communication skills can reduce inappropriate antibiotic prescribing, which could help to tackle antibiotic resistance. General practitioners often overestimate patient expectations for an antibiotic. In this study, we describe how general practitioners and patients with respiratory tract infections (RTI) communicate about their problem, including the reason for encounter and ideas, concerns, and expectations (ICE), and how this relates to (non-)antibiotic prescribing in out-of-hours (OOH) primary care.

**Methods:** A qualitative descriptive framework analysis of video-recorded consultations during OOH primary care focusing on doctor-patient communication.

**Results:** We analyzed 77 videos from 19 general practitioners. General practitioners using patient-centered communication skills received more information on the perspective of the patients on the illness period. For some patients, the reason for the encounter was motivated by their belief that a general practitioner (GP) visit will alter the course of their illness. The ideas, concerns, and expectations often remained implicit, but the concerns were expressed by the choice of words, tone of voice, repetition of words, etc. Delayed prescribing was sometimes used to respond to implicit patient expectations for an antibiotic. Patients accepted a non-antibiotic management plan well.

**Conclusion:** Not addressing the ICE of patients, or their reason to consult the GP OOH, could drive assumptions about patient expectations for antibiotics early on and antibiotic prescribing later in the consultation.

## Introduction

There is a growing problem of antibiotic resistance because of antibiotic overprescribing. Therefore, enhancing antibiotic prescribing quality is essential. Antibiotics are one of the most prescribed medications in out-of-hours (OOH) primary care, mainly for respiratory tract infections (RTIs) (1). In Belgian OOH care, there is antibiotic overprescribing (e.g., over 75% of patients presenting with tonsillitis and over 50% with bronchitis, sinusitis, and otitis media have been prescribed an antibiotic) and the overuse of non-guideline recommended antibiotics, which is in line with primary care during office hours (1). Also, in Norway and Sweden, there are no large differences during and outside office hours (2, 3). In the Netherlands, higher prescribing rates in OOH primary care were found, but with equal or even better quality compared with during office hours (4). In Iceland, a substantial number of patients with acute otitis media or pneumonia are prescribed broad-spectrum antibiotics in OOH primary care (5). In the United Kingdom, an increase in antibiotic prescribing outside compared with during office was seen in primary care (6, 7). And also in Danish OOH, primary care, antibiotics are the most prescribed medications (8). Although many contacts during OOH consist of self-limiting RTIs, and general practitioners (GPs) feel that most contacts represent non-urgent problems, they struggle to prescribe antibiotics prudently (9, 10). Out-of-hours GPs see unknown patients in a time-pressured setting that lacks follow-up possibilities or diagnostic tools (11, 12).

The problem presentation of patients and the communication of GPs affect antibiotic prescribing (13–16). The problem presentation refers to the communication at the start of the consultation where patients present their problems, including the reason for the encounter and the ideas, concerns, and expectations (ICE) of the patients. It is initiated by an opening question, and typically part of the “agenda mapping” phase of the consultation, which normally ends when history taking starts (17–20). Agenda mapping is essential in patient-centered communication (21, 22). Considering the agenda of both the doctor and the patient and taking time to elicit the problems and concerns of the patient, leads to higher patient satisfaction, reduces late arising concerns, improves understanding, time management, treatment adherence, and better health outcomes (20, 23–26). In practice, addressing the ICE of the patient does not always feel natural for GPs (27). Using ICE needs to be approached delicately, context-sensitive and patient-specific. It has been part of the medical training of Belgian GPs for many years now.

The way patients present their problem has been shown to be a predictor of receiving an antibiotic, such as using a candidate diagnosis (13, 15, 28) or discussing or justifying the need for a consultation (29, 30). GPs make assumptions about why the patient consults (31), and often overestimate patient expectations for antibiotics (28, 32). Research has shown that patients with an infection consult because of unpleasant symptoms, the perceived need for action, obtaining symptomatic treatment, or concerns about illness severity. Also, the need for assessment, information, or reassurance rather than expectations for antibiotics, are possible reasons to consult (33–35). Most expect a thorough examination and explanation for their symptoms (36). Soliciting patient expectations could lead to higher patient satisfaction and reduced antibiotic prescribing (37–39). But often, the patients do not voice their agenda, therefore keeping their needs hidden. One of the reasons is doctors quickly interrupting patients at the start of the consultation (40–45) or a lack of solicitation of this agenda (46). A Korean study in emergency care has shown that patients and their accompanying person do not only describe the problem but also discuss the reasonableness in their reason for encounter (47).

Despite a comparable quality of antibiotic prescribing during and outside office hours, GPs experience the decision to prescribe antibiotics differently in the OOH context. What remains unclear is how patients with RTIs communicate their reason to consult OOH primary care, how GPs and patients communicate about ICE and how this relates to (non-)antibiotic prescribing management and communication. A better understanding would enable to tailor future interventions to improve the antibiotic prescribing quality in this specific context.

Therefore, this study sets out to describe how patients present their problem and how GPs interact with this and elicit the ICE of patients, and in what possible ways this doctor-patient communication relates to antibiotic prescribing for RTIs in OOH care.

## Methods

### Study Context

This study is part of the Better Antibiotic prescribing through Action Research (BAbAR) project, which uses participatory action research (PAR) to improve the quality of the antibiotic prescribing of GPs in the Belgian OOH primary care (48).

Videos were recorded during the day at weekends from the end of August until November 2018 in the Antwerp city GPs cooperative (GPC), Belgium. In this GPC, all 185 GPs of the region are involved in a rotation system in one location during all weekends and bank holidays and cover a population of about 186,000 patients.

### Study Design, Participants, and Data Collection

Patients presenting in the OOH setting with infections were informed and invited by a medical student to participate. A small web camera was used, pointing at the GP and not the patient. The voices of the patients were captured. The GP was in charge of the camera. The researcher was not present in the consultation room. A maximum variation sample of GPs was used, to have a variety in sex and age. The GPs who were scheduled to work during the study period were invited to participate by email or telephone and received two cinema tickets as compensation. The GPs did not receive any additional communication training for this study. The GP, patient, and consultation characteristics were collected.

Some of the videos were used in a previous study for the purpose of elicitation interviews (31) and the complete study setup is described in a separate paper (49).

### Data Analysis

All videos were transcribed verbatim. Where applicable, Jeffersonian transcription, as shown in Table 1, was used to capture more detail on distinctive elements such as computer use, pauses, emphasis, and non-verbal signs (50). We used Excel (Microsoft Corporation, Washington, United States) to support the data analysis.

**Table 1:** Used symbols (derived from Jeffersonian transcription).

**Underlined text**	**Emphasizing**
(.)	Micropause usually <0.2 s
(# seconds)	The time, in seconds, of a pause
=	Continuation of speak
((text))	Annotation of non-verbal activity
::	Prolongation
><	Speeded talk

We used a combined deductive and inductive framework analysis with a descriptive approach (51). The analysis started with two data sessions with all authors to familiarize and emerge themselves in the data. In these sessions, 10% of the videos, with a range of cases (different GPs, different infections, antibiotics prescribed or not) purposefully selected by AC, were viewed to decide upon which recurring patterns of interaction would further lead the analysis. The team decided to investigate the problem presentation and how the GPs elicited the ICE concerning (non-)antibiotic prescribing for RTIs.

Then, an inductive approach was used to code 15% of the videos in sessions involving AC, SA, and KB (inductive component) (AC: GP; SA: social scientist, specialism: antibiotic prescribing behavior change; KB: GP, specialism: doctor-patient communication).

Subsequently, a framework was developed to organize the large amount of data based on the literature and the initial inductive analyses. It included parts of the Maastricht History-taking and Advice Scoring list consisting of global items (Maas global) and Calgary-Cambridge Guides (15, 23, 52, 53). The MAAS-global and Calgary Cambridge Guides are instruments to obtain objective measures to rate, feedback, and judge doctor-patient communication. The framework is provided as Supplementary Material 1. The relevant elements of the consultation were charted by AC (deductive component), and notes, apparent communication skills, and interpretations were added.

From this framework, AC, SA, and KB inductively identified the relevant results and discussed and described the final results. In the discussion, we have interpreted our results and discussed the potential implications of our results on antibiotic prescribing.

### Ethics

The study was approved by the Ethics Committee of the Antwerp University Hospital/University of Antwerp (reference number 17/08/089) and registered at ClinicalTrials.gov (NCT03082521). Permission for the video recordings was obtained from the Belgian Committee of Health of the Commission for the Protection of Privacy (SCSZG/18/067).

All the participating GPs and patients, who were eligible (presenting with an infection) and able (sufficient knowledge of the language), provided written informed consent. For patients under the age of 16 years, one of the parents or the legal guardian gave consent.

## Results

Out of 196 eligible patients with all types of infections, 152 (78%) agreed to participate. For this study, 77 videos on RTIs from 19 GPs were included (exclusion of consultations on skin infections, urinary tract infections, genital infections, and gastroenteritis). More details are presented in Table 2.

**Table 2:** Characteristics of the general practitioners (GPs), patients, and consultations.

**GP characteristics**	
Number of participating GPs	19
**Age in years of GPs**	
Mean (SD)	42.47 (13.41)
Median	39
Range (min-max)	26–64
**Years in practice**	
Mean (SD)	14.7 (12.42)
Median	12
Range (min-max)	1–38
**Gender distribution of GPs**	
Male	7 (37%)
Female	12 (63%)
**Type of GP practice they work in during regular office hours (outside OOH care)**	
Solo	2 (10.5%)
Duo	1 (5.3%)
Group	15 (78.9%)
Community health centre	1 (5.3%)
GP trainee (GP in specialty training)	2 (/19)
**Duration of the consultations in hh:mm:ss**	
Mean (SD)	00:12:19 (00:05:13)
Median	00:11:21
Range (min-max)	00:04:24–00:30:04
**Consultations per GP**	
Mean (SD)	4.05 (2.25)
Median	4
Range (min-max)	1–8
**Patient characteristics**	
Number of participating patients	77
**Age in years of patients**	
Mean (SD)	22.30 (21.75)
Median	22
Range (min-max)	0–89
Missing values	6
**Gender distribution of patients**	
Male	33 (43%)
Female	44 (57%)
**Consultations**	
**Different diagnoses**	
Upper respiratory tract infection	31
Otitis media/Otitis externa	10
Sore throat/pharyngitis/tonsillitis/uvulitis/throat abscess	13
Sinusitis	7
Viral/flu-like illness	5
Tracheitis/laryngitis	4
Bronchitis	3
Pneumonia	1
Bronchiolitis	1
Lymphadenopathy	1
Fever	1
Antibiotics prescribed	14 (4 delayed prescriptions) (18%)

*GPs, general practitioners; OOH, out-of-hours*.

We withheld three themes. Theme 1 shows how patients presented their reason to consult the OOH GP and how the GP elicited their perspective. Theme 2 shows how the concerns of the patients were expressed and/or handled. Theme 3 shows the lack of eliciting patient expectations and how delayed prescribing was used to accommodate expectations. Finally, theme 4 illustrates how the GPs communicated their treatment plan.

### Problem Presentation of the Patients

In the first phase of the consultation, we looked at how the patients presented their reason to seek help during the weekend and how the GPs explored the ICE of the patients.

#### Reasons of the Patient to Contact the GP on Call

After the GP opened the consultation, most patients started by listing their symptoms and/or suggesting a diagnosis. This diagnosis could be a viral [“uh I have the flu and I would like euhm, you know, get some medication eventually” (GP15, female, 36y - P57, female, 23y, upper respiratory tract infection (URTI))] or bacterial diagnosis [“well, I thought: do I have pneumonia?” (GP3, female, 63y, P10, female, 67y, pneumonia)], or even multiple diagnoses [“I'm really flu-like and I have a serious bronchitis” (GP6, male, 50y- P21, male, 31y, URTI)]. The suggested diagnosis sounded more like a label or vernacular name that patients used to show familiarity with these types of symptoms than an understanding of whether the cause was viral or bacterial.

Most patients spontaneously specified and legitimized the reason for the encounter at the start of the consultation. Patients set the context that influenced their decision to consult, such as going on holidays, work obligations, need for a sick note, being referred by a worried family member/partner, or the unavailability of their regular GP. Sometimes, but not always, these reasons implicitly contained the reason why the patient consulted OOH. Additionally, reasons related to the illness, such as worsening of symptoms, asking for medication, or being worried about a symptom were observed. Some examples are shown in Table 3. Often, the introductory talk already indicated some of the underlying ICE of the patients, but these were often not explicitly mentioned by the patient nor explored by the GP. When delivering the diagnosis, most GPs referred back to the (assumed) ICEs.

**Table 3:** Reasons to visit and possible ideas, concerns, and expectations (ICEs) during out-of-hours (OOH) care.

**Reasons to visit the OOH**	**Example**	**Other reasons or ICEs prompted in the consultation by the patient**
Leaving for vacation/holiday /work next day	P: the reason I come now instead of waiting until Monday is because I have to fly on Monday so I want to know if I should or not… (GP6, male, 50y- P21, male, 31y, URTI)	P: I'm really flu-like and I have a serious bronchitis. … I think it's because of the air conditioning in the airplane. … I don't often take medication. It has to be really bad then. … Last time I had this kind of cough, I let it go on too long and then I came to the doctor and he said I had a bad pneumonia. So I didn't want that this time. GP: ok, and it was cured with? P: yes, just with some antibiotics, and then it was better … I just came back from the States, yes busy life … and then I'm getting married … GP: And a jet lag? P: a jet lag, yes, but I can handle that pretty well … If I can work? I work with clients, one on one, because I don't want to make anyone else sick
Sent by family member/partner	P: well I think I've got one of these typical airway infections but my wife said if you can't swallow properly you have to have it checked out, to see it's not an angina (GP16, female, 28y - P61, male, 38y, sore throat)	P: I have taken paracetamol, it helps against the muscle strain, headache, but not against the pain when I swallow, … It started on Friday, and now it's Sunday, I still suffer from it … paracetamol makes it bearable, but if I don't take it I almost can't swallow. … During clinical examination: -I recently took antibiotics for a tick bite. So I think my body is just too weak in general
Double consultation as two people in the household are ill	P: so I thought I will go anyway (0.4) I will let him get checked and then me because I feel even more ill (GP11, female, 39y - P36 male, 3y, URTI)	P: I can't swallow well, even my own saliva hurts, and also when I'm eating and drinking … and I'm pregnant too
Regular GP not available/ Need of a sick note	P: I have been in bed with the flu for a few days, but I have to work tomorrow, I'm not up for it, and in the Netherlands they are very accommodating but in Belgium… …. P: My GP couldn't see me anymore today (0.2) so I thought … (GP8, female, 28y - P26, male, 21y, URTI)	During clinical examination: P: yes, I've also had mononucleosis GP: yes? P: actually a year now … P: and I also have the idea that it is a bit more difficult in terms of immunity
Worsening of symptoms	Mother: It has been a few days now that he's got an airway infection and now this morning he said this night he really was in pain (GP16, female, 28y - P60, male, 6y, otitis)	- M: when he complains, something is really going on - euh and we will soon leave for Legoland so that's why I eum - yes I just wanted to check
Asking for medication	P: I went to the pharmacy (.) three days ago GP: oh yes (looks at the medication) P: and I picked up this but it's not really working GP: no? P: it's more like candy (GP13, female, 29y - P46, male, 74y, tracheitis/laryngitis)	P: I think I have a throat infection - but I also have a strong cough, less during the day now, but at night and in the morning I really have to cough a lot
Worried about a symptom	Mother: what should we do? because I see a lot of white spots in her throat (GP17, female, 51y - P64, female, 26y, sore throat)	P: It's an awful lot of pain when I swallow and talk - I went to work yesterday, it was ok, but around noon I took some pain-killers because I was so in so much pain - But I am not someone who takes painkillers quickly, I will say thatM: now our grandson normally comes tomorrow,what would we do? P: And then I also had antibiotics and since then I have been terrified about these. The following week I had sores all over my mouth.
To have a thorough clinical examination	Mother: yes, she had a heavy cold just recently so the question is to examine her again thoroughly (GP13, female, 29y - P44, female, <1y, URTI)	M: 3 days ago she got her vaccinations as well - She has been going to day care for a while and she has had such a strong cold several times, with a cough. And then, um, for her nose, yeah, what was that actually called
		but for the day care they only wanted to give it, if you have a prescription. - and I had already been to the doctor twice, and the doctor said by phone: yes, give her the drops - especially whiny, but it is not too bad. She's a really super happy baby, so During clinical examination: She is ok on the curve they said. But she is, she only weighs 5.2 kilos or so

*AB, antibiotics; RTI, respiratory tract infection; OOH, out-of-hours; P, patient; M, mother; GP, general practitioner; y, years; URTI, upper respiratory tract infection; RSV, respiratory syncytial virus*.

In only two out of the 77 consultations, two different GPs explicitly elicited the reason why the patient decided to consult the GP on call during the weekend.

The way some patients formulated their reason for encounter sounded as if visiting the GP would alter the course of their illness. For example, a patient expressed the idea that by going to the doctor (and expecting a treatment?), they would prevent the illness from getting worse. Some patients have had a previous experience of a serious infection and were told that they were “too late” or “just in time” so implying that timing of consultation in the course of illness has an important impact on the evolution of the illness. Table 4 shows some examples.

**Table 4:** Examples of patients who imply that visiting their GP will alter the course of their illness.

**Quote**
P: I don't have any fever now but I think tonight I will have a seriously high fever (GP13, female, 29y, P47, male, 48y, viral/flu-like illness)
P: a lot of snot a bit of cough GP: a little cough? P: a little (GP starts typing) P: nothing else (0.3) no (0.5) GP: yes P: but in the past Euh 2 years ago I had a pneumoniaso that's why (GP18, female, 46y, P70, female, 40y, URTI)
P: I guess It's all not that bad but I thought, if I don't go now, it probably will get worse and mostly the ear bothers me (GP11, female, 39y - P37, female, 42y, URTI)
P: and I thought euhm I will go to the practice, here because I'm afraid it could become a throat infection euhm, but I don't make any fever so(GP15, female, 36y -P55, male, adult, tracheitis/laryngitis)
GP: Do you have health issues we should know of? Of the respiratory tract? P: No. Last time I had this kind of cough, I let it go on too long and then I came to the doctor and he said I had a bad pneumonia. So I didn't want that this time. GP: how long ago was it? P: three, 4 years ago, something like that GP: ok, and it was solved with? P: yes, just with some antibiotics, and then it became better. (GP6, male, 50y - P21, male, 31y,URTI)

*P, patient; GP, general practitioner; y, years URTI, upper respiratory tract infection*.

#### How GPs Elicit the Perspective of the Patients

There were differences between GPs in how much time they gave patients for their problem presentation. The GPs with an empathic open attitude, who gave space to talk and present the problem, who made eye contact and did not immediately start typing, who picked up cues and asked open questions and made encouraging sounds, spontaneously received a lot of information about the ICE of the patients. Some GPs explicitly elicited the ICE at the start of the consultation. But also in these consultations, we still observed other ICEs popping up later in the consultation, for example during the clinical examination or the management phase.

Two discrepant consultation styles showed one GP who created plenty of space for the perspective of the patients and a second GP who often interrupted the patient without attention to the agenda of the patient. The open communication and understanding of the concerns and expectations of the patient in the former GP, led, in one case, to a (most likely) unnecessary delayed antibiotic prescription (also see Delayed Prescribing to Meet Patient Expectations?). The latter GP both asked closed questions and completed the history taking much faster. She also communicated her immediate decision not to prescribe antibiotics much more directly to the patient, not leaving any space for the patient to protest. We added more detailed analysis of this interaction and comprehensive excerpts of these two different GP styles as Supplementary Material 2.

### How GPs Address the Concerns of Patients

Patients did not always explicitly mention concerns nor did the GP elicit them. Only two GPs actively probed if the patient was concerned, of which one GP routinely asked it during all her consultations. The GPs addressed possible concerns throughout the clinical examination by commenting out loud on what they saw/heard or not and explained why this reassured or worried them. Often, GPs summarized their clinical findings when explaining the diagnosis and used them to explain why they did or did not prescribe antibiotics. They explained to the patient that they were not worried, for example, because the lungs sounded clear, the ears were not that red, the throat looked ok, and so on, that it looked like a viral infection, and there was no need for antibiotics. If they did prescribe an antibiotic, they expressed their concerns about certain symptoms and justified the antibiotic prescription.

If the GP did not address the concerns of the patients, the patients often mentioned them again later on in the consultation. We noticed that not addressing the concerns made it more difficult for the GP to “defend” a non-antibiotic management strategy. In the following example, there was an indirect resistance by a mother, who was concerned about her baby girl with fever and cough. The GP tried to reassure her by saying that it was “just an airway infection.” However, after this fragment, the GP acknowledged that children could be very ill from a viral infection, she gave safety-netting advice and explained why she is not worried and at that point, the mother accepted the treatment plan.

**Table:** 

*GP: I don't think this is something we should give antibiotics for*	*GP delivers the diagnosis with a non-antibiotic treatment plan*.
*I think if we give antibiotics I don't expect it will get better spectacularlyI don't expect that at all*. *(GP starts typing)* *GP: So I really think it's just (0.5) an airway infection* *and that you have to sweat it out*	*The GP uses the term “just an airway infection”* and there is no treatment necessary, which seems to minimize the symptoms.
*Mother: It looks so heavy then*. *(GP frowns)* *Mother: but if you don't see anything like that, well* *…* *Mother to the child (smirky tone): well (.) you're completely fine then*	*The mother shows resistance. She uses the word “well” and expresses this by saying in a sarcastic tone that there is nothing wrong with you to the child*.
*GP: No, no, she does have an infection, there is indeed something wrong* *Mother: yes because she has a feverGP: yes, indeed, but not everyone makes high fever, there are people with the same infection who will not have 39 degrees fever.* *Mother: yes*	*The GP acknowledges the mother in the fact that the child is sick. She tries to give reassurance about the fever*.
*And uh uh* *When should I go back to the doctor or already* *-within a day or two?* *GP: if she still has a fever on Tuesday I would have her checked by a doctor again, to check her ear* *Mother: yes* *GP: if that has gotten worse um* *If pus runs out of the ear(.)* *I would also take her to the doctor* *If- if her intake is not ok, if feeding and drinking does not go well* *If she's getting short of breath* *Mother: yes* *GP: < but at this point we see nothing like that>* *Mother: °no*	*The mother asks about when there is a need to reconsult. The GP gives detailed safety netting advice*.
*Mother: but that cough-* *GP: yes (doctor nods), but her lungs sound fine, so I'm not worried*	*The mother still expresses some worrying about a symptom. GP uses clinical findings to reassure her*.
*Mother: ok, thank you* *GP: so keep an eye on herbut you are doing well*	*Acceptance by the mother. GP acknowledges the mother she is doing fine and gives some last advice*.
*> be sure to rinse her nose so there is as little pressure as possible on the ear <*	
*But otherwise* *Mother: yes yes yes*	

*(GP5, female, 38y - P14, female, 2y, URTI)*.

### Expectations

We explored how GPs and patients communicated about what help the patient expected for the problem. Clear patient expectations led to smooth and straightforward consultations. And we observed how delayed prescribing was used to meet patient expectations for antibiotics.

#### Clear Patient Expectations and Straightforward Consultation

In general, GPs nor patients in this study talked directly about their expectations from the consultation and none of the GPs elicited the view of the patients about receiving antibiotics. Asking if the patient had to work that day and if they needed a sick note, was commonly used as an indirect way to clarify the expectations of the visit of the patients. Several patients spontaneously explained to the GP that they needed a sick note and that this was their reason for the encounter. This is generally followed by a straightforward consultation without any prescriptions.

#### Delayed Prescribing to Meet Patient Expectations?

Four patients received a delayed antibiotic prescription. In two cases, the GP prescribed the antibiotic after the patient questioned the non-antibiotic decision explicitly. These patients used different strategies to negotiate an antibiotic prescription. One patient prepared the antibiotic prescription by presenting himself as a person who would not just take antibiotics if it is not necessary. He said he was “not a big fan of antibiotics” and “he had received a delayed prescription in the past and did not collect it.” Although the GP first clearly explained why antibiotics are not necessary, he issued a delayed prescription after the patient questioned his decision (“But no antibiotics?”). We included an excerpt of this consultation and discuss the observed interaction in Supplementary Material 2.

The other patient used the argument that in her experience her regular GP does not offer an antibiotic prescription at the first consult for her child, but that she always needs to go back as the child gets worse without antibiotics.

### Non-antibiotic Management Plan

After the clinical examination, the GP communicated the diagnosis and a treatment plan.

#### Non-antibiotic Management Plan Well-Accepted

In most cases, the GP gave the diagnosis after the clinical examination, and this statement was immediately followed by a non-antibiotic management plan. In many cases, the GPs literally indicated that there was no indication for antibiotics. Often this information was combined with some reassuring elements of the clinical examination or the absence of alarm symptoms, such as in the following excerpt.

**Table:** 

GP: it actually all looks goodP: yes	GP refers to the findings of the clinical examination
GP: sorry, that's very frustrating but sometimes things like this can take 2 weeksP: yes	GP normalizes the duration of the complaints
GP: it is still-for me- no indication for antibiotics	GP indicates that antibiotics are not necessary
it's also better for your own body to overcome this.hhP: yes	GP arguments why antibiotics are not good
GP: but I would definitely do, is rinse your nose and use a nasal spray because-	GP offers a symptomatic treatment plan

*(GP2, female, 26y, P6, female, 27y, URTI)*.

In limited cases, the patients asked if they needed an antibiotic immediately after the GP explained the diagnosis. When a GP clarified why antibiotics were not needed, most patients accepted this decision as shown in the following consultation.

**Table:** 

P: so the diagnosis is? GP: yes, I think it's just a viral infection	Patient asks what the diagnosis is. GP gives a viral diagnosis.
P: yes, a lot of people GP: yes, there are a lot of people flu-ish this week. Well okay, it's not the flu yet, but feverish- P: yes I am too, it really feels like, cold-warm, cold-warm all the time. I don't know, I'm sweating so hard. So, yes, that's how I feel	Patient recognizes the symptoms.
GP: yes, yes. But you can't do more than recuperate a bit. You already have, you have all the home remedies that you need. Euhm, I can't really add, euhm, yeah, take it easy for a few days	GP indicates that rest is the best option and confirms that the patient's current symptomatic treatment is adequate
P: but definitely no antibiotics?	Patient questions if she needs antibiotics.
GP: no, it looks very much like a viral infection. That's also how your throat looks, huh: your throat looks very red P: ah ok	GP arguments why antibiotics are not necessary and links it with the physical examination. Patient accepts.

*(GP11, female, 39y, P37, female, 42y, URTI)*.

In only one out of the 77 consultations, the non-antibiotic treatment plan evoked a conflict with a patient, but the GP did not budge. In this consultation an expectation for an antibiotic was not explicitly explored by the GP, but could have been assumed in the following interaction:

GP: and you're here because you say the medication doesn't work that well?

P: no no you need to give me good medication

Cause I know that-

that if it helps (.) it can't get any worse (0.6)

if it works, the medicine

After the physical examination, the patient clearly indicated that he expected and needed an antibiotic. Although the GP at that point used different communication techniques (reflection of feelings, acknowledging the seriousness of the complaints, explaining why antibiotics are not necessary, providing a symptomatic alternative, and so on) to persuade the patient, the resistance lasted until the end of the consultation. The patient and GP in this case had different ethnic roots. So possibly, this consultation was complicated by cultural differences and the use of a non-native language by both the GP and the patient. We provided a more comprehensive excerpt and analysis of the interaction of this consultation in Supplementary Material 3.

Many GPs offered non-antibiotic management wrapped in the narrative “it is a viral infection, antibiotics won't help,” which was well-accepted by most patients. In one case a patient replied he did not expect an antibiotic, in another, a patient talked about a previous serious side-effect of the antibiotics and would prefer not to take antibiotics. In the following example, parents consulted the GP before traveling to India because they did not want to receive antibiotics unnecessarily and they had more trust in the Belgian doctors.

*GP: I don't know if you have euh over there you have the possibility*

*of medical*=

*Father:* =*yes yes, we have we have*

*I just think the only problem in India is that the doctors give*

*antibiotics too quickly*.

*GP: ah ha*

*Father: and I, yeah, we don't want to do so*

*that's my only concern*

*and that's why we always bring our child to the doctor here*

*(GP9, male, 42y - P27, male, 3y, URTI)*

#### Discussing the Treatment Plan

Sometimes “antibiotics” were not explicitly mentioned, but GPs used phrases such as: “We can't do anything” or “not to use a cannon (to kill a flea),” as shown in the examples in Table 5.

**Table 5:** How GPs explain self-limiting disease/medication is not necessary.

GP: so yes, you have a throat infection again euh::m (.) it's probably because of a virus P: mmhh GP: you can't do a lot .hh if a had a little wonder pill I would have given it to you …. there is no point of giving antibiotics, it's not a bacterial infection, you do not have a fever. P: yes (GP2, female, 26y - P9, female, 19y, URTI)
GP: but he has an infection of his upper airways … GP but we can't always go on bombing with canons if it's not necessary. … GP: so sometimes if we find microbes we do prescribe some heavier medication but if it's not necessary….well (GP7, male, 58y - P22, female, 4y, URTI)
P: so what now? GP: yes, I think you need some more patience I can't do anything preventively (GP15, female, 36y - P55, male, adult, tracheitis/laryngitis)
GP: often (.) almost always it's a viral infection what is annoying? that we can't do a lot you have to sit it out. but that's also the positive (.) there's no harm (GP14, male, 56y - P49, female, 11y, sore throat)

*P, patient; GP,general practitioner; y, years*.

The GPs addressed expectations about medication prescriptions when they were discussing the treatment plan. If certain undiscussed expectations were not met, patients would bring these up themselves. For example: “no coughing syrup?,” “no throat spray?” Some GPs discussed these expectations and the needs of the patients before prescribing, and they explained the reasons for not choosing a certain treatment.

## Discussion

In this study, we examined how patients with RTI present their complaints to OOH primary care and how GPs interact with them. Patients with RTIs mostly start a consultation by listing symptoms and/or suggesting a possible diagnosis (28). Stivers et al. showed that parents from children using a bacterial candidate diagnosis instead of a description of their symptoms were more likely indexed by the physician as expecting an antibiotic, but Cabral et al. found that parents rarely implied an expectation for antibiotics for children with RTI and there was no relationship with the antibiotic prescribing of GPs. Globally, the trend of patient expectation for receiving antibiotics for RTIs is declining (54), but still, the problem of overprescribing remains due to many patient- and doctor-related reasons, such as lack of time, pleasing, perceived severity, and so on but also due to the health care system and the overall culture (55–57).

Patients give different reasons for their decision to consult the GP during OOH. This could be interpreted as a justification or legitimations for the consultation, in the literature also described as “doctorability” (18). Almost none of the GPs elicit specifically why the patients choose to consult OOH (this could reveal a lot of the ICE of the patients) keeping the actual reason for the encounter implicit. They do not actively ask patients about their views on antibiotics and seldom explore other expectations. Eliciting patient expectations for an antibiotic has been proposed as an intervention to reduce inappropriate antibiotic prescribing (58, 59). Other studies have shown that expectations are seldom directly discussed (60, 61). We found that exploring the ICE of patients is not commonly done for RTIs in Belgian OOH primary care. Conversely, Lemiengre et al. have shown that eliciting parental concerns can potentially increase antibiotic prescribing (62). Indeed, making space for the agenda of the patients, also creates a potential pitfall when empathy is confused with yielding to inappropriate treatment decisions in response to very present ICE. This can lead to unnecessary antibiotic prescriptions, as we have illustrated with a few deviant cases. The MAAS Global manual explicitly states that the GP's indulgence in content is not a characteristic of empathy (53).

Research shows that active listening and addressing the ICE of patients leads to higher patient satisfaction and fewer follow-up consultations (22). But there is often an infrequency in soliciting the full range of the concerns of patients (63), as we could confirm in our study. (Re)consultation is one of the high-risk factors of receiving an antibiotic, and therefore, optimizing doctor-patient communication could affect antibiotic prescribing. Addressing the health belief that a GP influences the evolution of infection and educating patients on the natural evolution of infections and self-treatment could have an impact on (re)consultations in the future as well. Especially in OOH primary care where non-urgent problems could better be seen by the regular GP.

The narrative “it's a viral infection, you don't need an antibiotic” confirms the dichotomized thinking of GPs during consultations of RTIs (bacterial vs. viral infection/antibiotics vs. no antibiotics) (31) and also ignores the fact that bacterial infection does not always benefit from antibiotics. GPs seem to assume that the reason a patient visits the GP on call with an RTI, amongst others, is receiving an antibiotic, by delivering the diagnosis directly linked to the non-antibiotic management plan, which is well-accepted by patients. At this point in the consultation—after the history taking and physical examination—GPs have sufficient arguments to back up their diagnosis and to argue whether antibiotics are necessary or not. It is recommended to elicit the ICE early in the consultation. But, without information from the physical examination, it is more difficult to discuss a possible antibiotic expectation already at that point. In an Irish study using questionnaires, one in three patients attending OOH with acute RTI symptoms expected to receive antibiotics before the consultation (34). Only patients hoping for antibiotics are less satisfied, when not prescribed an antibiotic (not the ones expecting or asking antibiotics) (64–67).

GPs who make room for the story of patients by using active listening techniques spontaneously receive more information about the ICE. Patients do not always explicitly talk about their concerns, but GPs use their findings of the clinical examination to reassure patients that there is no reason to give antibiotics. Some GPs ask patients what they need or what symptoms bother them the most and show a nice example of shared decision-making in prescribing symptomatic medication or self-treatment. A refocus from “we can't do anything” to informed shared decision-making about what the patient needs, could help (68, 69). Other communication tools, such as safety-netting, could be helpful in communicating when to (re)visit a GP and educating patients for following episodes. Also, triage and postponed care to the regular GP during office hours could lead to fewer RTIs presented to OOH primary care (10).

We saw that assumed or expressed expectations for antibiotics can lead to a delayed antibiotic prescription even though antibiotics were not clinically necessary. Delayed prescribing has been proposed as an intervention to reduce antibiotic prescribing (70). GPs in one interview study, talked about the use of delayed prescribing as a way not to jeopardize the doctor-patient relationship (71). In another interview study, it is shown that different GPs use delayed antibiotic prescribing in different ways, one of which is maintaining a good relationship to retain patients (72). But in an OOH setting, with no long-term relationship with the patient, this argument should play less. An established trusted relationship, which you do not have in OOH primary care, could help to use delayed prescriptions because the GP can estimate if a patient will follow the instruction of the GP and has sufficient understanding and if the GP can transfer the final decision onto the patient (72). In a previous interview study in the OOH primary care context, elements such as pleasing, reciprocity or time-pressure also played a role in the antibiotic prescribing decision (11).

### Strengths and Limitations

Our video recordings captured real-life GP behavior and doctor-patient communication. It enabled us to collect rich relevant data from a broad range of different GPs, patients, and RTIs. The use of this type of data collection is rather recent and not yet applied on a large scale, contributing substantially to research on this topic. We used well-established communication models as a framework to analyze the consultations. The MAAS global has been proven effective in communication skills training to lead to more prudent prescribing of antibiotics for URTIs (73).

Triangulation with researchers from different backgrounds enhanced trustworthiness. To assess transferability, we clearly described our participants and the context in which this study took place, namely Belgian OOH primary care, which is a high prescribing context, with a fee-for-service system and no triage.

The GPs and patients were aware of the fact that antibiotic prescribing was our focus, and this could have influenced their communication and prescribing behavior, the so-called Hawthorne effect. Although GPs fed back that their recorded consultations reflected their normal consultation style (49), our participants could be low prescribers adhering more to the guidelines. Therefore, what we observed could be best practices. We could not objectively judge if a decision to prescribe an antibiotic was medically justified. Possibly some patients declined to participate because they were afraid not to receive antibiotics. All these elements could explain the rather low antibiotic prescribing rate we observed. Although this limitation must be recognized, it did not impede the study of communicative patterns which was the focus of the analysis.

Unfortunately, we were not able to analyze the non-verbal communication of the patients (49). We also performed a more explorative analysis. Other analyses, such as conversation analysis, could lead to more in-depth knowledge of certain patterns of interaction.

This study was performed before the COVID-19 pandemic. The meaning of the phrase “it's just a viral infection, you don't need to worry” has changed dramatically and could nowadays have a different effect on patients.

## Conclusion

This study in OOH primary care shows that the actual reason for encounters for a patient with an RTI often remains implicit. However, when giving empathic attention to the story of the patients, many of the ICE of the patients appear spontaneously and this could help to tailor the communication about non-antibiotic decisions, educate patients about the self-limiting aspect of RTIs, and to improve patient satisfaction. Delayed antibiotic prescribing is sometimes used to meet the patient expectation for antibiotics and to safeguard the doctor-patient relationship. In clinical practice, attention to high-quality doctor-patient communication when explaining the diagnosis and treatment plan seems as important as exploring the reason for encounter and ICE to reduce antibiotic prescribing. Communication skills training has been proven to improve antibiotic prescribing in primary care during office hours. Future research could focus on implementing communication strategies to enhance prudent antibiotic prescribing for RTIs in OOH primary care.

## Data Availability Statement

The datasets presented in this article are not readily available because the data are not publicly available due to restrictions, it contains information that could compromise the privacy of our research participants.

## Ethics Statement

The studies involving human participants were reviewed and approved by Ethics Committee of the Antwerp University Hospital/University of Antwerp. Written informed consent to participate in this study was signed by the participating GPs, the patients and for children by the legal guardian.

## Author Contributions

AC: conceptualization, data curation, formal analysis, investigation, methodology, project administration, writing—original draft, review, and editing. KB: formal analysis, methodology, writing—review, and editing. HP: supervision, writing—review, and editing. RR and SC: supervision, funding acquisition, writing—review, and editing. SA: conceptualization, formal analysis, methodology, supervision, writing—review, and editing. All authors contributed to the article and approved the submitted version.

## Funding

This study was part of the BAbAR study and has been granted a PhD fellowship by the Faculty of Medicine and Health Sciences of the University of Antwerp. The funder had no involvement in any of the research stages.

## Conflict of Interest

The authors declare that the research was conducted in the absence of any commercial or financial relationships that could be construed as a potential conflict of interest.

## Publisher's Note

All claims expressed in this article are solely those of the authors and do not necessarily represent those of their affiliated organizations, or those of the publisher, the editors and the reviewers. Any product that may be evaluated in this article, or claim that may be made by its manufacturer, is not guaranteed or endorsed by the publisher.

## Supplementary Material

Data Sheet 1 Supplementary Material 3. Conflict about non-antibiotic decision.

Data Sheet 2 Supplementary Material 2. How GPs elicit the patients' perspective: Two different approaches.

Data Sheet 3 Supplementary Material 1. Analysis framework.

## References

[1] Colliers A Adriaenssens N Anthierens S Bartholomeeusen S Philips H Remmen R. Antibiotic prescribing quality in out-of-hours primary care and critical appraisal of disease-specific quality indicators. Antibiotics. (2019) 8:79. 10.3390/antibiotics8020079 31212871 PMC6628021

[2] Cronberg O Tyrstrup M Ekblom K Hedin K. Diagnosis-linked antibiotic prescribing in Swedish primary care-a comparison between in-hours and out-of-hours. BMC Infect Dis. (2020) 20:1–11. 10.1186/s12879-020-05334-7 32819280 PMC7441551

[3] Lindberg BH Gjelstad S Foshaug M Høye S. Antibiotic prescribing for acute respiratory tract infections in Norwegian primary care out-of-hours service. Scand J Primary Health Care. (2017) 35:178–85. 10.1080/02813432.2017.1333301 28569649 PMC5499318

[4] Debets VE Verheij TJ van der Velden AW. Antibiotic prescribing during office hours and out-of-hours: a comparison of quality and quantity in primary care in the Netherlands. Br J Gene Practice. (2017) 67:e178–86. 10.3399/bjgp17X689641 28232364 PMC5325659

[5] Palsdottir HA Jonsson JS Sigurdsson EL. Prescriptions of antibiotics in out-of-hours primary care setting in Reykjavik capital area. Scand J Primary Health Care. (2020) 38:265–71. 10.1080/02813432.2020.1794159 32672085 PMC7470112

[6] Hayward GN Fisher R Spence G Lasserson D. Increase in antibiotic prescriptions in out-of-hours primary care in contrast to in-hours primary care prescriptions: service evaluation in a population of 600 000 patients. J Antimicrob Chemother. (2016) 71:2612–9. 10.1093/jac/dkw189 27287234 PMC4992853

[7] Edelstein M Agbebiyi A Ashiru-Oredope D. Trends and patterns in antibiotic prescribing among out-of-hours primary care providers in England, 2010-14. J Antimicrob Chemother. (2017) 72:3490–5. 10.1093/jac/dkx323 28961983 PMC5890685

[8] Christensen MB Nørøxe KB Moth G Vedsted P Huibers L. Drug prescriptions in Danish out-of-hours primary care: a 1-yearpopulation-based study. Scand J Primary Health Care. (2016) 34:453–8. 10.1080/02813432.2016.1248622 27804314 PMC5217277

[9] Keizer E Smits M Peters Y Huibers L Giesen P Wensing M. Contacts with out-of-hours primary care for nonurgent problems: patients' beliefs or deficiencies in healthcare? BMC Family Practice. (2015) 16:157. 10.1186/s12875-015-0376-9 26510620 PMC4625560

[10] Smits M Colliers A Jansen T Remmen R Bartholomeeusen S Verheij R. Examining differences in out-of-hours primary care use in Belgium and the Netherlands: a cross-sectional study. Eur J Public Health-Oxford. (2019) 29:1018–24. 10.1093/eurpub/ckz083 31086964 PMC6896980

[11] Colliers A Coenen S Remmen R Philips H Anthierens S. How do general practitioners and pharmacists experience antibiotic use in out-of-hours primary care? An exploratory qualitative interview study to inform a participatory action research project. BMJ Open. (2018) 8:e023154. 10.1136/bmjopen-2018-023154 30269072 PMC6169767

[12] Williams SJ Halls AV Tonkin-Crine S Moore MV Latter SE. General practitioner and nurse prescriber experiences of prescribing antibiotics for respiratory tract infections in UK primary care out-of-hours services (the UNITE study). J Antimicrobial Chemother. (2018) 73:795–803. 10.1093/jac/dkx429 29190384 PMC5890663

[13] Scott JG Cohen D Dicicco-Bloom B Orzano AJ Jaen CR Crabtree BF. Antibiotic use in acute respiratory infections and the ways patients pressure physicians for a prescription. J Family Pract. (2001) 50:853. 11674887

[14] Stivers T. Participating in decisions about treatment: overt parent pressure for antibiotic medication in pediatric encounters. Soc Sci Med. (2002) 54:1111–30. 10.1016/S0277-9536(01)00085-5 11999506

[15] Stivers T Mangione-Smith R Elliott MN McDonald L Heritage J. Why do physicians think parents expect antibiotics? What parents report vs what physicians believe. J Family Pract. (2003) 52:140–7. 12585992

[16] Zhou Y MacGeorge EL Hackman N. Parent-provider communication and antibiotic prescribing for pediatric ear infections. Commun Res Rep. (2019) 36:170–8. 10.1080/08824096.2019.1586666

[17] Heritage J Robinson JD. The structure of patients' presenting concerns: physicians' opening questions. Health Commun. (2006) 19:89–102. 10.1207/s15327027hc1902_1 16548700

[18] Heritage J Robinson JD. Accounting for the visit: Giving reasons for seeking medical care. Stud Int Sociolinguistics. (2006) 20:48. 10.1017/CBO9780511607172.00530886898

[19] Robinson JD. An interactional structure of medical activities during acute visits and its implications for patients' participation. Health Commun. (2003) 15:27–59. 10.1207/S15327027HC1501_2 12553776

[20] Gobat N Kinnersley P Gregory J.W, Robling M. What is agenda setting in the clinical encounter? Consensus from literature review and expert consultation. Patient Educ Counsel. (2015) 98:822–9. 10.1016/j.pec.2015.03.024 25892504

[21] Norfolk T Birdi K Walsh D. The role of empathy in establishing rapport in the consultation: a new model. Med Educ. (2007) 41:690–7. 10.1111/j.1365-2923.2007.02789.x 17614890

[22] Silverman J Kurtz S Draper J. Skills for Communicating With Patients. New York, NY: CRC Press (2016). 10.1201/9781910227268

[23] Kurtz SM Silverman JD. The Calgary-Cambridge Referenced Observation Guides: an aid to defining the curriculum and organizing the teaching in communication training programmes. Med Educ. (1996) 30:83–9. 10.1111/j.1365-2923.1996.tb00724.x 8736242

[24] Wanzer MB Booth-Butterfield M Gruber K. Perceptions of health care providers' communication: relationships between patient-centered communication and satisfaction. Health Commun. (2004) 16:363–84. 10.1207/S15327027HC1603_6 15265756

[25] King A Hoppe RB. “Best practice” for patient-centered communication: a narrative review. J Graduate Med Educ. (2013) 5:385. 10.4300/JGME-D-13-00072.1 24404300 PMC3771166

[26] Robinson JH Callister LC Berry JA Dearing KA. Patient-centered care and adherence: Definitions and applications to improve outcomes. J Am Acad Nurse Pract. (2008) 20:600–7. 10.1111/j.1745-7599.2008.00360.x 19120591

[27] Spence D. Falling through the ICE. BMJ. (2009) 338:b5. 10.1136/bmj.b5

[28] Stivers T. Presenting the problem in pediatric encounters: “Symptoms only” versus “candidate diagnosis” presentations. Health Commun. (2002) 14:299–338. 10.1207/S15327027HC1403_2 12186491

[29] Rollnick S Seale C Rees M Butler C Kinnersley P Anderson L. Inside the routine general practice consultation: an observational study of consultations for sore throats. Family Practice. (2001) 18:506–10. 10.1093/fampra/18.5.506 11604372

[30] Stivers TJ. Negotiating Antibiotic Treatment in Pediatric Care: The Communication of Preferences in Physician-Parent Interaction. Los Angeles, CA: University of California (2000).

[31] Colliers A Coenen S Bombeke K Remmen R Philips H Anthierens S. Understanding general practitioners' antibiotic prescribing decisions in out-of-hours primary care: a video-elicitation interview study. Antibiotics. (2020) 9:115. 10.3390/antibiotics9030115 32156082 PMC7148451

[32] O'Doherty J Leader LF O'Regan A Dunne C Puthoopparambil SJ O'Connor R. Over prescribing of antibiotics for acute respiratory tract infections; a qualitative study to explore Irish general practitioners' perspectives. BMC Family Pract. (2019) 20:27. 10.1186/s12875-019-0917-8 30764777 PMC6374900

[33] Huibers L Carlsen AH Moth G Christensen HC Riddervold IS Christensen MB. Patient motives for contacting out-of-hours care in Denmark: a cross-sectional study. BMC Emerg Med. (2020) 20:20. 10.1186/s12873-020-00312-3 32183705 PMC7079359

[34] O'Connor R O'Doherty J O'Regan A O'Neill A McMahon C Dunne CP. Medical management of acute upper respiratory infections in an urban primary care out-of-hours facility: cross-sectional study of patient presentations and expectations. BMJ Open. (2019) 9:e025396. 10.1136/bmjopen-2018-025396 30772860 PMC6398638

[35] O'Connor R O'Doherty J O'Regan A Dunne C. Antibiotic use for acute respiratory tract infections (ARTI) in primary care; what factors affect prescribing and why is it important? A narrative review. Irish J Med Sci. (2018) 187:969–86. 10.1007/s11845-018-1774-5 29532292 PMC6209023

[36] Mortazhejri S Patey AM Stacey D Bhatia RS Abdulla A Grimshaw JM. Understanding determinants of patients' decisions to attend their family physician and to take antibiotics for upper respiratory tract infections: a qualitative descriptive study. BMC Family Pract. (2020) 21:1–11. 10.1186/s12875-020-01196-9 32580696 PMC7313109

[37] Little P Stuart B Francis N Douglas E Tonkin-Crine S Anthierens S. Antibiotic prescribing for acute respiratory tract infections 12 months after communication and CRP training: a randomized trial. Ann Family Med. (2019) 17:125–32. 10.1370/afm.2356 30858255 PMC6411389

[38] Cabral C Ingram J Lucas PJ Redmond NM Kai J Hay AD. Influence of clinical communication on parents' antibiotic expectations for children with respiratory tract infections. Ann Family Med. (2016) 14:141–7. 10.1370/afm.1892 26951589 PMC4781517

[39] McNulty CA Nichols T French DP Joshi P Butler CC. Expectations for consultations and antibiotics for respiratory tract infection in primary care: the RTI clinical iceberg. Br J Gen Pract. (2013) 63:e429–36. 10.3399/bjgp13X669149 23834879 PMC3693799

[40] Barry CA Bradley CP Britten N Stevenson FA Barber N. Patients' unvoiced agendas in general practice consultations: qualitative study. BMJ. (2000) 320:1246–50. 10.1136/bmj.320.7244.1246 10797036 PMC27368

[41] Britten N Stevenson FA Barry CA Barber N Bradley CP. Misunderstandings in prescribing decisions in general practice: qualitative study. BMJ. (2000) 320:484–8. 10.1136/bmj.320.7233.484 10678863 PMC27293

[42] Kravitz RL Cope DW Bhrany V Leake B. Internal medicine patients' expectations for care during office visits. J Gene Internal Med. (1994) 9:75–81. 10.1007/BF02600205 8164081

[43] Bell RA Kravitz RL Thom D Krupat E Azari R. Unsaid but not forgotten: patients' unvoiced desires in office visits. Arch Internal Med. (2001) 161:1977–84. 10.1001/archinte.161.16.1977 11525700

[44] Peltenburg M Fischer JE Bahrs O van Dulmen S Van den Brink-Muinen A. The unexpected in primary care: a multicenter study on the emergence of unvoiced patient agenda. Ann Family Med. (2004) 2:534–40. 10.1370/afm.241 15576537 PMC1466741

[45] Marvel MK Epstein RM Flowers K Beckman HB. Soliciting the patient's agenda: have we improved? JAMA. (1999) 281:283–7. 10.1001/jama.281.3.283 9918487

[46] Dyche L Swiderski D. The effect of physician solicitation approaches on ability to identify patient concerns. J Gene Internal Med. (2005) 20:267–70. 10.1111/j.1525-1497.2005.40266.x 15836531 PMC1490080

[47] Lee S-H Kim CW. Presentation of patients' problems during triage in emergency medicine. Patient Educ Counsel. (2015) 98:578–87. 10.1016/j.pec.2015.01.011 25665739

[48] Colliers A Coenen S Philips H Remmen R Anthierens S. Optimising the quality of antibiotic prescribing in out-of-hours primary care in Belgium: a study protocol for an action research project. BMJ Open. (2017) 7:e017522. 10.1136/bmjopen-2017-017522 29038184 PMC5652575

[49] Colliers A Coenen S Remmen R Philips H Anthierens S. Looking inside the out-of-hours primary care consultation: general practitioners' and researchers' experiences of using video observations as a method. Int J Qualit Methods. (2019) 18:1609406919879341. 10.1177/1609406919879341

[50] Jefferson G. Transcript notation. Struct Soc Action. (1984) 5:346–69. 10.1017/CBO9780511665868.02130886898

[51] Gale NK Heath G Cameron E Rashid S Redwood S. Using the framework method for the analysis of qualitative data in multi-disciplinary health research. BMC Med Res Methodol. (2013) 13:1–8. 10.1186/1471-2288-13-117 24047204 PMC3848812

[52] Cabral C Horwood J Symonds J Ingram J Lucas PJ Redmond NM. Understanding the influence of parent-clinician communication on antibiotic prescribing for children with respiratory tract infections in primary care: a qualitative observational study using a conversation analysis approach. BMC Family Practice. (2019) 20:102. 10.1186/s12875-019-0993-9 31324157 PMC6642577

[53] van Thiel J Ram P van Dalen J. MAAS-Global Manual. Maastricht: Maastricht University (2000).

[54] Kianmehr H Sabounchi NS Seyedzadeh Sabounchi S Cosler LE. Patient expectation trends on receiving antibiotic prescriptions for respiratory tract infections: A systematic review and meta-regression analysis. Int J Clin Practice. (2019) 73:e13360. 10.1111/ijcp.13360 31066959

[55] Borek AJ Anthierens S Allison R Mcnulty CA. Social and contextual influences on antibiotic prescribing and antimicrobial stewardship: a qualitative study with clinical commissioning group and general practice professionals. Antibiotics. (2020) 9:859. 10.3390/antibiotics9120859 33271843 PMC7759918

[56] Touboul-Lundgren P Jensen S Drai J Lindbæk M. Identification of cultural determinants of antibiotic use cited in primary care in Europe: a mixed research synthesis study of integrated design Culture is all around us. BMC Public Health. (2015) 15:1–9. 10.1186/s12889-015-2254-8 26381376 PMC4574721

[57] Tonkin-Crine S Yardley L Little P. Antibiotic prescribing for acute respiratory tract infections in primary care: a systematic review and meta-ethnography. J Antimicrob Chemother. (2011) 66:2215–23. 10.1093/jac/dkr279 21764827

[58] Cals JW Butler CC Hopstaken RM Hood K Dinant GJ. Effect of point of care testing for C reactive protein and training in communication skills on antibiotic use in lower respiratory tract infections: cluster randomised trial. BMJ. (2009) 338:b1374. 10.1136/bmj.b1374 19416992 PMC2677640

[59] Little P Stuart B Francis N Douglas E Tonkin-Crine S Anthierens S. Effects of internet-based training on antibiotic prescribing rates for acute respiratory-tract infections: a multinational, cluster, randomised, factorial, controlled trial. Lancet. (2013) 382:1175–82. 10.1016/S0140-6736(13)60994-0 23915885 PMC3807804

[60] Altiner A Knauf A Moebes J Sielk M Wilm S. Acute cough: a qualitative analysis of how GPs manage the consultation when patients explicitly or implicitly expect antibiotic prescriptions. Family Practice. (2004) 21:500–6. 10.1093/fampra/cmh505 15367471

[61] Mustafa M Wood F Butler CC Elwyn G. Managing expectations of antibiotics for upper respiratory tract infections: a qualitative study. Ann Family Med. (2014) 12:29–36. 10.1370/afm.1583 24445101 PMC3896536

[62] Lemiengre MB Verbakel JY Colman R De Burghgraeve T Buntinx F Aertgeerts B. Reducing inappropriate antibiotic prescribing for children in primary care: a cluster randomised controlled trial of two interventions. Br J Gene Practice. (2018) 68:e204–10. 10.3399/bjgp18X695033 29440016 PMC5819986

[63] Robinson JD Tate A Heritage J. Agenda-setting revisited: When and how do primary-care physicians solicit patients' additional concerns? Patient Educ Counsel. (2016) 99:718–23. 10.1016/j.pec.2015.12.009 26733124

[64] Welschen I Kuyvenhoven M Hoes A Verheij T. Antibiotics for acute respiratory tract symptoms: patients' expectations, GPs' management and patient satisfaction. Family Practice. (2004) 21:234–7. 10.1093/fampra/cmh303 15128681

[65] Coenen S Francis N Kelly M Hood K Nuttall J Little P. Are patient views about antibiotics related to clinician perceptions, management and outcome? A multi-country study in outpatients with acute cough. PLoS ONE. (2013) 8:e76691. 10.1371/journal.pone.0076691 24194845 PMC3806785

[66] Coenen S Van Royen P Vermeire E Hermann I Denekens J. Antibiotics for coughing in general practice: a qualitative decision analysis. Family Practice. (2000) 17:380–5. 10.1093/fampra/17.5.380 11021895

[67] Coenen S Michiels B Renard D Denekens J Van Royen P. Antibiotic prescribing for acute cough: the effect of perceived patient demand. Br J Gene Pract. (2006) 56:183–90. 16536958 PMC1828261

[68] Land V Parry R Seymour J. Communication practices that encourage and constrain shared decision making in health-care encounters: Systematic review of conversation analytic research. Health Expect. (2017) 20:1228–47. 10.1111/hex.12557 28520201 PMC5690232

[69] Coxeter P Del Mar CB McGregor L Beller EM Hoffmann TC. Interventions to facilitate shared decision making to address antibiotic use for acute respiratory infections in primary care. Cochrane Database Syst Rev. (2015) 2015:CD010907. 10.1002/14651858.CD010907.pub2 26560888 PMC6464273

[70] Spurling GK Del Mar CB Dooley L Foxlee R Farley R. Delayed antibiotic prescriptions for respiratory infections. Cochrane Database Syst Rev. (2017) 9:CD004417. 10.1002/14651858.CD004417.pub5 28881007 PMC6372405

[71] van der Zande MM Dembinsky M Aresi G van Staa TP. General practitioners' accounts of negotiating antibiotic prescribing decisions with patients: a qualitative study on what influences antibiotic prescribing in low, medium and high prescribing practices. BMC Family Pract. (2019) 20:1–11. 10.1186/s12875-019-1065-x 31823739 PMC6905031

[72] Saliba-Gustafsson EA Röing M Borg MA Rosales-Klintz S Lundborg CS. General practitioners' perceptions of delayed antibiotic prescription for respiratory tract infections: A phenomenographic study. PLoS ONE. (2019) 14:e0225506. 10.1371/journal.pone.0225506 31756197 PMC6874332

[73] Strumann C Steinhaeuser J Emcke T Sönnichsen A Goetz K. Communication training and the prescribing pattern of antibiotic prescription in primary health care. PLoS ONE. (2020) 15:e0233345. 10.1371/journal.pone.0233345 32428012 PMC7237035

