# Supplementary material for: Antibiotic Prescribing and Doctor-Patient Communication During Consultations for Respiratory Tract Infections: A Video Observation Study in Out-of-Hours Primary Care
Source: Front Med (Lausanne). 2021 Dec 1;8:735276. doi: 10.3389/fmed.2021.735276 (PMC8671733; doi:10.3389/fmed.2021.735276)
Supplement: Data Sheet 1 — Supplementary Material 3. Conflict about non-antibiotic decision. [file Data_Sheet_1.PDF]

Supplementary material 3: Conflict about non-antibiotic decision

(GP10, female, 27y, P31, male, 61y, Viral/flu-like illness)

GP: hmm<br>P: uh so uh the day before yesterday so the doctor he =<br>GP: =yes:: ↑<br>P: gave this medicine =<br>..<br>GP: Okay so tell me<br>P: okay so I'm shivering from the cold [now]<br>GP: [yes]<br><br>*history taking*	GP types on the computer, in between turns to patient, so switching between eye contact and computer writing.
P: and so I took those medicines↑<br>GP: when did you go to the doctor?<br>P: yesterday<br>GP: and when did it all start?<br>P: yesterday!<br>GP: yesterday… and tell me apart from this shivering…<br>P: I feel terrible, stiff, …	Nodding, use of encouraging words 'yes', summarizing
GP: muscle pains…<br>P: yeah here and here…but before I did not have such heavy pains, when I took the medication….<br>GP: but I will look in a minute to the medication that your doctor prescribed. So you shiver from the cold and have muscle pains, any other complaints?<br>P: …<br>GP: I will ask a few questions now, do you have a headache?<br>P: yes, also<br>GP: ear pain?	Open questioning, non-verbal encouraging sounds, screening, summarizing, echo, agenda setting
P: no	GP shakes head
GP: throat pain?	GP nods head
P: no only when I cough<br>GP: so you do cough…<br>P: yes<br>GP: nose obstruction?<br>P: it seems obstructed<br>GP: diarrhea or vomiting?<br>P: no but stomach complaints<br><br>GP: o you got [medication]<br>P: [but that didn't work either]<br>GP: it didn't help<br>P: no, no	

more history taking<br><br>GP: and you're here because you say the medication doesn't work that well?<br>P: no no you need to give me good medication 'Cause I know that-<br>that if it helps (.) it can't get any worse (0.6)<br>if it works, the medicine<br>GP: depends a bit on what the cause is huh<br>But you have the feeling that-<br>that it is not sufficient [for you ?<br>P: [no, no I'm getting tired of it]<br>…<br><br>*doctor uses computer: 00:02:40-00:04:00 while the patients talks about his situation and the symptoms a bit more*	GP questions the reason for contact.<br>Patient indicates that he needs strong medication<br>Combined typing on the computer, and eye-contact in between.<br><br>GP checks with patient.
Start physical examination 00:04:13<br><br>GP: do you have other important medical problems?<br>P: no<br>GP: any medication you need to take apart from those pills?<br>P: no<br>GP: your throat looks a bit red	*GP asks some more question about the symptoms while examining, and explains what she does and what she finds, which is mostly reassuring.<br>GP gives verbal instructions during examination.<br>*end of physical examination: 00:07:25*
P: I've had it before<br>GP: yes<br>P: but that's uhm a long time ago<br>Maybe my immunity has gotten better huh<br>GP: yes yes	
P: but eu::h (.) then in my country I got antibiotics<br>GP: antibiotics yes<br>P: so that it passes quickly. over there you always do (.)<br>For example, if you have a cold<br>There is a lot of difference in the weather (.)<br>Different circumstances than here<br>GP: [yes yes]<br>P: if you catch a cold (.)<br>GP: yes<br>P: they prescribe antibiotics quickly<br>Yes Yes<br>P: and I can't wait anymore (.)<br>Because it's very dangerous isn't it (0.4)<br>that virus huh	GP sits down, starts typing.<br>GP uses echo<br>Again switching between eye contact and computer.

GP: yes, ok, now<br>You've got a very strong virus, haven't you?<br>P: [yes pff]<br>GP: you are very sick<br>P: .hhhh<br>GP: you are shivering (0.5) you had a high fever yesterday (0.3)<br>Now it has gone down a bit because of the medication (.) huh<br>P: yay<br>GP: but it's a virus isn't it<br>P: yes<br>GP: it is a flu-like virus (0.3)<br>P: yes<br>GP: Eh:m (.)<br>Now antibiotics (.) won't help<br>Here in Belgium we do not prescribe antibiotics (0.4)<br>For virus infections (.)<br>It's something that will go away by itself (.)<br>You are going to have to rest several days (0.3) huh<br>P: hmm<br>GP: rest well<br>P: hmm<br>GP: you can take paracetamol<br>[three to four times a day]<br>P: [but euh euh I get eu::h (0.2) bad headaches from them]<br>GP: normally the headache should improve with that (0.3) huh<br>P: no this morning also one (.) and it doesn't help<br>P: It’s not the right medicine (0.4)<br>…<br>*doctor uses computer: 00:09:00-00:09:40*<br>…	GP stops typing and turns to patient, uses gestures.<br><br>Acknowledges the seriousness of the symptoms and summarizes.<br><br>GP diagnoses a viral infection.<br><br>GP explains why antibiotics won’t work. No Chunck & Check, little space is provided for questions, the patient nevertheless does interrupt the GP and asks his questions<br><br>GP tells what he can do.<br><br>GP leans forward, with the hands on the table, starts writing a prescription.<br><br>Patient resists the GP’s decision.<br>GP suggests to prescribe stronger pain medication and why it should work.
P: and (.) why not an antibiotic?<br>GP: om-<br>Because that won't help (0.8)<br>it's a virus<br>P: in my country you always get (0.3) one of those cures<br>What's it called eu:h (.)<br>That's a kind of (0.9) yes (0.4) omentin?<br>GP: augmentin (=amoxiclav) yes<br>Now you're going to get sick much faster next time aren't you<br>If we start giving antibiotics now<br>It suppresses [your immune system	GP starts typing on the computer.<br><br>GP repeats the diagnosis.<br><br>GP gives an extra reason why she does not prescribe antibiotics.

P: [yes I know but I can hardly walk now]<br>GP: I'm sorry that you have such a strong muscle pain<br>P: a lot of pain!<br>GP: that's why I'm going to give a heavier painkiller (0.6)<br>Do you need a note for work? (0.6)<br>…<br>*Explains how to use the painkillers*	GP turns to the patient.<br><br>GP closes the discussion by switching the topic to the sick note. She remains talking calm, clear and loud, same tempo, no hesitations, eye contact.
GP: If you get sicker (0.6) huh<br>If u uh (.) can no longer eat or drink (0.4)<br>Then you should let it checked again<br>P: I can't sit here honestly<br>I can't sit here normally<br>I need to get well soon<br>Really uh (.) you have to write out antibiotics quickly<br>Because really (0.4) the age and stuff, that's different isn't it<br>GP: [I know I know]<br>P: young people and stuff, they can handle that well<br>But I'm older something like uh (0.3) 60 huh<br>GP: hmm<br>P: I'm not going to let it run its course because you know (.)<br>I want to get well soon (.) huh =<br>GP: = I understand, I understand<br>P: because it's very bad<br>GP: hmm hmm yes<br>P: yes you haven't experienced it yet yes hhhhhhh<br>GP: now (0.4) I understand that you (.) you feel very bad (0.2)<br>But you are not helped with antibiotics at the moment (0.3)<br>So we won't write it (0.4)<br>Eu::hm =<br>P: = yes you're not gonna write it (0.4)<br>So I have to come back then<br>GP: yes next week for a check-up with your GP<br>…	GP gives safety netting advice.<br><br>Patient reopens the discussion about not getting antibiotics.<br><br>GP nods, eye contact, listens.<br><br>Empathic support.<br><br>Reflection of feelings.<br><br>GP show understanding attitude but sticks to her decision, uses 'we' to make her statement more powerful.<br><br>GP repeats her message. (joining and downing)
GP: Antibiotics at this time are not going to help you<br>I know it's hard and you feel sick<br>But (.) that's all I can do for you right now<br>P: no (4.0)<br>Better to go to my own GP (5.3)	GP repeats her message.

repeats symptomatic treatment and closes consultation	
GP types a lot during beginning of the consultation which gives a closed body position, at the end during the discussion she turns more to the patient and does have an open body posture, non-verbally encouraging, repetitions and paraphrasing, agenda setting, room for patients' perspective in the beginning, sticks to her decision not to prescribe antibiotics despite the patient's resistance, explains why, first confirms patient's symptoms, offers alternative symptomatic medication.	Length 00:12:28
Time that patient can tell his story after opening question before first interruption of GP (anamnestic question)	15 seconds
MAAS Global scores (by experienced comm skills professor)	Item 1 (introduction, 0-6): 2<br>Item 3 (request for help, 0-6): 3
