# Supplementary material for: Antibiotic Prescribing and Doctor-Patient Communication During Consultations for Respiratory Tract Infections: A Video Observation Study in Out-of-Hours Primary Care
Source: Front Med (Lausanne). 2021 Dec 1;8:735276. doi: 10.3389/fmed.2021.735276 (PMC8671733; doi:10.3389/fmed.2021.735276)
Supplement: Data Sheet 2 — Supplementary Material 2. How GPs elicit the patients' perspective: Two different approaches. [file Data_Sheet_2.PDF]

Supplementary material 2.

Examples of 3.1.2 : How GPs elicit the patients' perspective: Two different approaches

Approach 1: Quick and straightforward approach

(GP11, female, 39y, P36, female, 37y, URTI)

GP: good morning<br>It's for you huh<br>P: it's [for] me and [for him]<br>GP: [yes] [tell]<br>P: I had [2 consultations]<br>GP: [yes,yes]	Opening the consultation<br>GP opens file on the computer<br>GP turns to the patients and nods. Eye contact.
P: eu:hm (0.4) I've had hhh(0.5) for four days now ↑ my throat hurts so much (0.5)<br>hh. But it's ::(.) now only [got] worse<br>GP: [hmmm]<br>P: so I thought I'm going to have that checked (0.4) for him and certainly also for me because I'm a bit sicker (0.3) =<br>GP: = yes (0.6)	Patients gives the reason why she came. Patient talks about duration and pain.<br>Two people sick lowered the threshold to come?<br>Worsening of symptoms.<br>Wants it to be checked.<br>GP encourages the patient non-verbally (eye-contact during 12sec, arms and hands open on the table, small verbal encouragements)
P: A::nd if (.) I can't [swallow] because even (0.4)<br>GP: [yes]<br>P: my own (0.4) saliva hurts [and] -<br>GP: [yes]<br>P: and also when I eat and drink =<br>GP: = yes (0.7)	Patient shows the seriousness of the sore throat.<br>GP turns to the computer.
GP: And do you have a fever? (0.6)<br>P: eu::hm I did measure it, but it is 38.9, 38 (0.7)<br>Eu:h it does not exceeds 39<br>GP: [no no no no] (0.6)	GP starts history taking. She is still looking at the computer. Closed questions.<br>Time between opening question from GP "Tell?" until interruption with first anamnestic question 'And do you have a fever'? = 20 sec
GP: okay (0.4) yes (0.3)<br>Cough a little? (0.5)<br>P: eu::hm (.) yes but not very [very::]<br>GP: [yes]<br>P: erm when I'm coughing [occasionally]<br>GP: [yes]<br>P: But is mostly real [drowsiness]<br>GP: [yes yes]<br>P: erm (.) headache ↑ (1.3) here and=<br>GP: =and how long do you have a fever? (1.0)<br>P: erm two days (1.6)<br>GP: okay that's good =	GP turns to the patient again. GP continues history taking while using lots of 'yes' as confirmation.<br>Closed suggestive question.<br>GP sets the tempo (rather speedy), no further clarifications of the symptoms, interrupts the patient who is talking.<br><br><br><br><br>GP interrupts the patient.<br>She turns to the computer again
GP: = And you are pregnant too =<br>P: = yes	GP turns to the patient again. Closed questions.

Yes I am [uh]<br>GP: [about how far?] (0.5)<br>P: I started this week (0.2) uh 7th month<br>GP: oh yes (0.3) okay<br>I'll check you first<br>maybe your uhm (0.3)<br>You can take off your clothes	Time from first anamnestic question until announcement of physical examination = 46sec.<br>GP gestures to take off clothes.
P: I had maybe only an hour or half an hour of sleep last night<br>GP: oh that's not good huh<br>P: no (1.1)<br>Crying a lot<br>Until 3 o clock then I went to sleep	*doctor uses computer: 00:01:28 – 00:01:52*<br>While patient goes to the examining table, the doctor starts typing. At the same time the patient tells about the bad night. Brief eye contact during 'Oh that's not good huh', GP smiles
Clinical examination<br>GP: open your mouth<br>P coughs<br>GP: low blood pressure, always?<br>P: …<br>GP: 9 on 6, that is low, this explains….<br>P: …<br>GP: certainly when you are tired, not sleeping well. Your throat is swollen so...<br>P: …<br>GP: No, your tonsils are not infected, it's the back of your throat,<br>P:…<br>GP: it's your lymph nodes that are swollen, yes. It's mainly a troth infection, the rest seems okay.<br>P:…<br>GP: Yes yes yes<br>P:…<br>GP: he has a fever as well?<br>P:… cough and fever is less now, nose obstructed, still now, but yesterday it was very… he almost doesn't sleep, very agitated, turning and turning and turning in his bed, and suddenly he was awake and crying a lot, seemed to have pain.<br>GP: come and sit here, you big boy. We will look into your ears. What ear is aching?<br>…<br>GP: Say Aaa. You can do this very well!<br>GP: Does he have diarrhea? Also many lymph nodes.<br>P: …<br>This ear does look a bit red. A little bit! He will feel it. But his throat looks well. I would not give him antibiotics for this. It is not really a big ear infection but only a little bit of pressure on his	Start physical examination: 00:01:52*<br>GP examines the mother, no verbal communication apart from instruction to open mouth until 00:04:30 = 'low blood pressure'. Interruptions of patient while explaining physical examination.<br><br>GP examines the child while doing history taking, mother underscores child is very agitated and crying a lot (sounds affected while saying so, could be a cue to concerns). GP does not explore concerns.<br><br>GP reassures the mother and advices about a symptomatic treatment for him. Patient asks questions but is constantly interrupted by GP, who does answer the questions but very briefly, with interruptions, high tempo of answers, no use of silence.

ear drum instead of a real infection. Keep the nose clear, that is the most important in fact. Does he drink enough? This is something that will heal spontaneously, yes yes.<br>P: no ear drops?<br>GP: no this is behind his ear drum so… better to use nose drops before he sleeps, yes.<br>P: …<br>GP: Use salty water or ear drops, this will help for his ear pain. Yes?<br>P: …<br>GP: It doesn't look bad, huh, but he will feel it. Certainly during the night, they are tired and can't sleep and…<br>But you should give the children a short while to someone else so that you can rest, or you will not get better, huh!	
	After physical examination
GP: Rest is the best medicine for you (5.1)<br>Are you at home or working? (0.3)<br>P: no I am currently [home yes]<br>GP: [currently] at home yes yes (1.3)<br>P: I am overstressed (0.3) I have been sick a lot<br>The past weeks<br>*unintelligible*<br>Only got my blood pressure checked by my GP<br>GP: yes<br>P: and it has dropped to 9.5(0.3)<br>GP: ah yes so 9 out of 6 is not that bad yes<br>Good<br>So he (the child) can use a nose spray (.)<br>And for you, you need to drink enough for that throat (1.2)<br>P: nothing=<br>GP: = yes =<br>P: soothing for (.) that (0.2) it gets worse<br>Because it's been three days now (1.8)<br>GP: yes but you are not allowed to have much during your pregnancy (1.3)<br>P: [pregnant yes]<br>GP:<br>For a swollen throat (0.4)<br>I often say, make ice cubes and it will numb (0.6) and helps the swelling (0.7)<br>P: and something to disinfect=<br>GP: =and tea: ↑ a little honey something like that or a little ginger in it<br>P: and for disinfecting or something?<br>GP: that's not necessary<br>That's not necessary it doesn't look that ugly (.)	GP sits back down and turns to the computer.<br><br>GP does not pick up this cue: No eye contact, eyes on computer.<br><br>Non verbally: nodding, eye contact<br><br>GP turns back to the patient. Shows non-verbally to drink enough.<br><br>Patients shows some resistance, and asks for something for the throat<br><br>GP advices home remedies and supports her advice with hand gestures and nodding, eye contact.<br><br>GP shakes the head.<br>Patient repeats her question for medication.

It's swollen and red but the throat looks not too bad<br>Just (0.5) yes drink a lot<br>A lot of swallowing is-<br>is even<br>That disinfection is really not necessary<br>I really wouldn't do that (.)<br>no (.) no (1.1)<br>P: okay<br>GP: yes if you need to take paracetamol you can also during the [pregnancy]<br>P: [yes]<br>GP: that's it actually<br>P: okay (1.1) let's hope it doesn't get any worse (3.1)<br>GP: yes<br>I think that's the best<br>Bye<br>P: bye	Refusal of the GP to prescribe any medication, and clarification why.<br><br><br><br><br><br><br><br><br><br><br>GP does not answer this cue while looking her in the eyes.<br><br><br>Patient accepts, with some hesitation.<br>GP nods.
Length of the consultation: (2 consultations combined: child and mother, the child's history taking is done after the physical examination of the mother while the GP examines the child)<br>Speedy consultation, no room for the patient's concerns or expectations.	0:12:17, history taking stops at 00:01:23
Time that patient can tell her story after opening question before first interruption of GP (anamnestic question)	20 seconds
MAAS Global scores (by experienced comm skills professor)	Item 1 (introduction, 0-6): 2<br>Item 3 (request for help, 0-6): 1
Patient goes home without any prescriptions.	

Approach 2: Space for the patients' perspective approach, and prescribes a delayed prescription

(GP9, male, 42y, P28, male, 52y, sinusitis)

Patient had come to the OOH center a few weeks before with a similar problem	
P: So I felt bad (0.9) my throat and a runny nose and so on<br>And- (0.4) and she asked me does your sinus hurt↑<br>No::, okay and also a little cough and a little this and that and (1.4)<br>she said yes okay that's a virus<br>>If you feel that it gets worse within 2 or 3 days (0.8)<br>And, and, and<br>Fever and what do I know<	GP turns to the patient. Eye contact during 60 sec while patient tells his story. He makes encouraging sounds, shows interest and listens attentively. Arms and hands in open posture.

And then she had already prescribed (0.6)<br>Eu::hm<br>antibiotics<br>ultimately it wasn't necessary<br>thank God ↑ (1.8)<br>And okay uhm yes it's still not gone<br>but it's certainly not worse<br>So I thought okay (0.5)<br>But then two days ago-<br>always in the weekend (1.1)<br>And then I started to feel a pressure here<br>But actually first to my teeth (0.8)<br>'Cause I know I have sensitive teeth<br>and I know that those sinuses (.)<br>sometimes I can feel it on those [teeth]<br>GP: [hmmm] [hmmm]<br>P: I mean I have a lot of pain in those teeth (1.3)<br>pressure (0.8)<br>And and last night<br>it's calmed down a bit now (0.7)<br>…	Patient had received an antibiotic prescription during a previous consultation. "Thank God" patient emphasizes he is happy he did not had to use the prescription.<br><br>GP smiles.<br><br>GP leans back. Nodding.
GP: When did that pain start here?<br>((points to his jaw))<br>P: eu::hm (.), yes 2- (.)<br>[2-3 days]<br>GP: [2-3 days]<br>P: > so Thursday, because Thursday I always think yes I go Friday-<br>I always say yes I'm going to the doctor on Friday, but actually I don't have a doctor but I need to find one < (1.2)<br>I took medication<br>And then went to sleep early, drank a lot<br>>I don't know if that's psychological or that's okay, but I woke up Friday and went to work< (1.0)<br>And after a night's sleep you always feel it's better of course ↑<br>GP: [hmmm]<br>P: and then probably when that pressure drops (0.8)<br>hey so i thought ok<br>And then (.) Friday (0.6)<br>And then Saturday, yesterday actually (1.1)<br>>I Felt and then last night<br>When I sleep I always lay with my face down<br>But then the pressure is of course worse< GP: [yes,yes] ((nods))<br>P: So I couldn't-<br>that was really (1.4)<br>I don't want to be petty<br>But (0.8) that really felt like a blockage	GP handgestures to his jaw.<br>Open history taking question after 67 sec from start of consultation. After this single question, patient continues his story without interruptions during 2min40sec.<br>GP remains eye-contact, does not use computer until 00:09:40 (after the physical examination)<br><br>Repetition.<br><br>Patient does not have a regular GP, which could make safety netting or referral more difficult.<br><br>Nodding. Encouraging sounds. Eye contact without interruption. Open posture, hands folded on the table.

Well (0.2) 10-15 years ago-<br>>Well, I've always had a bit of sensitive airways(0.5)<br>so actually (1.0) that's how it was <<br>GP: [ahh]<br>yes =<br>P: = Throat infections, little bit of ear problems, (0.3) sno:: t (0.4)<br>GP: [yes]<br>P: But that was it<br>…	Patient emphasizes the severity of the pain<br>Patients is talkative. GP gives lots of space for this mainly by non-verbal active listening techniques.
Patient talks about a previous similar episode where he went to the ENT doctor	GP listens attentively, does not interrupt the patient, apart from 2 short clarification questions supporting the patients story.
GP: Have you taken anything in the past few days?<br>P: no:: because I have nothing =<br>GP: = no<br>>We grew up in a family<br>When you were sick we went to the doctor<br>and he prescribed what he had to prescribe<br>GP: yes<br>P: >we bought exactly that (.) it helped and then on we go again<<br>And (.)<br>We did not go buy things at the pharmacy ourselves<br>GP: [yes, yes, yes] ((nods))<br>P: like >Ah we took that last time, so we'll take that again<br>We always went to the doctor< (1.1)<br>If I have to take a paracetamol, then I will take it<br>But I'm like<br>GP: [yes not too much medication on its own]<br>P: [yes, kind of part of the healing process] and then you know (.)<br>And then you experience that a little bit<br>>not like that then you have to take this and this<<br>GP: yes yes yes (1.1)<br>GP: pretty good philosophy (0.4)<br>°yes (0.4)	GP still has an open listening attitude. Nodding.<br>Patient emphasizes that he trusts what the doctor prescribes and will not just take medication himself. GP non-verbally acknowledges that he understood this well.<br>GP paraphrases.<br>GP acknowledges the patient in its approach
…	More history taking.
Physical examination	Start physical examination: 00:04:35-<br>During the physical examination, the patient shows interest in the GP's own practice and asks if he can become a regular patient of the GP. The GP answers his office doesn't accept new patients at this moment, but recommends another 'good' medical office in the neighborhood.
GP: Your lungs sound **clear**.	The GP constantly announces what he will examine

P: my previous doctor said I only came just in time, it was just not a bronchitis…<br>…<br>GP: your ears are not infected, but it is normal when there is something going on in the ENT area that it radiates…<br><br>GP: so it is actually an inflammation of the sinus, while your nose is rather clear. This is because the opening from your nose to your sinus is obstructed. What the man (in your story) did, with a needle, we do not do that, that’s a specialists’ job, but what we can certainly try<br>…	GP delivers the diagnosis at the examining table.
GP: What we can certainly try, and what I would recommend to you is actually eu::hm(0.9)<br>Use a nasal spray to just open that opening=	At the examining table the GP delivers a non antibiotic treatment.
P: =but no antibiotics?<br>Isn't that necessary? It’s not that I say, I really want antibiotics but I really want to get rid of the pressure.<br>GP: yes (.) **normally** such a spray does open it up, it will effect that [pressure really] (.)<br>P: [take away yes]<br>GP: take away yes<br>So **standard** if there is no fever or if it is not longer than a week with pain<br>we don't write antibiotics<br>P: no<br>GP: **Sometimes**, uhm<br>>But that’s rather the exception, if it doesn't work with that spray<<br>Then, we do prescribe them (0.4)<br>Eu:::hm<br>…	Patient promptly asks if antibiotics are necessary?<br><br>GP uses the words: “standard”, and “normally”, which could be seen as introduction for an exception.<br><br><br><br><br><br><br>Feels if GP is not going to write antibiotics.
GP: do you still have that prescription lying around?<br>P: no<br>would you maybe<br>I'll start with that spray<br>GP: [yes that's good]<br>P: I'm not a big fan of antibiotics<br>GP: no, the fact that you didn't use the previous one<br>P: voila<br>GP: no that's good	GP asks about the prescription of the previous consultation.<br><br>Patient asks to rewrite it.<br><br>GP says he will write an antibiotic prescription.<br>Patient tries to gain trust from the GP, (which he also did during the history taking?)<br>GP confirms this trust.
GP: we're Sunday now,<br>Sunday, Monday Tuesday<br>So I think you should give it until Tuesday with that spray (0.3)<br>And-	GP explains when to use the delayed prescription.

and actually (.) it usually works<br>P: yes okay<br>GP: and if that’s not the case<br>Or you get a fever on top<br>P: yes<br>GP: okay then –<br>then go<br>P: no okay yes<br>GP: shall we do it like this?	
GP: euhm, did you have to work today?<br>P: no<br>GP: tomorrow?<br>P: no, so I'm self-employed<br>GP: ah yes	GP sits back down at the desk and starts typing and writing.<br><br><br>((GP works on computer 00:09:53-00:10:15))
P: I always wonder that you, who care for so many ill persons, that you don’t get ill?	Some small talk.<br>GP takes the time to make his prescription. Non-verbally he seems concentrated and calm/peaceful, only doing one thing at the time (listening to the patient OR taking care of the computer and prescribing, with great emphasis on the former)<br>GP explains how to take the antibiotics.<br>Patient asks and gets a prescription for his chronical medication (which in Belgium is regularly not done during OOH care)<br>Closing of the consultation
Lots of room during the history taking for the patient’s perspective, takes about 4 minutes, non-verbal encouragement, listening, open body posture, little boundaries with talkative person, lots of room for the patient’s ideas, concerns hearable, expectations not questioned, but still come up in the form of an antibiotic prescription at the end?	0:13:34
Time that patient can tell his story after opening question before first interruption of GP (brief anamnestic question)	67 sec + 2min40 sec
MAAS Global scores (by experienced comm skills professor)	Item 1 (introduction, 0-6): 6<br>Item 3 (request for help, 0-6): 5
Patient goes home with a delayed antibiotic prescription.	
