# Supplementary material for: Antibiotic Prescribing and Doctor-Patient Communication During Consultations for Respiratory Tract Infections: A Video Observation Study in Out-of-Hours Primary Care
Source: Front Med (Lausanne). 2021 Dec 1;8:735276. doi: 10.3389/fmed.2021.735276 (PMC8671733; doi:10.3389/fmed.2021.735276)
Supplement: Data Sheet 3 — Supplementary Material 1. Analysis framework. [file Data_Sheet_3.PDF]

Supplementary material 1. Analysis framework.

Exploring requests for help	Score	quote(s)	What communication triggered it? Spontaneous? Initiated by doctor? By what question? By findings clinical examination?,…	At what point in the consultation? (1=Establishing reason, 2=gathering information, 2a: anamnesis, 2b physical examination, 3=delivering diagnosis, 4=treatment planning, 5= closing, 6=unclear, 7=other, specify)	Is it picked up?<br><br>Is something done with it? How?	Is the link made with prescribing antibiotics or not?<br><br>Is there an effect on prescribing behaviour? How?	Other remarks<br><br>Striking details? Something important missing? Effective communicative elements? Non-verbal signs? What worked? What didn't work?, …
Reason for the visit/problem presentation							
REQUEST FOR HELP (item 3 MAAS: score 0-6)<br>0 = not present /1 = poor /2 = unsatisfactory /3 = doubtful<br>4 = satisfactory/ 5 = good /6 = excellent							
1. patient's ideas (i.e. beliefs re cause)<br>What are the patient's suspicions or assumptions with regard to the cause of the complaint or problem.							
2. patient's concerns (i.e. worries) regarding each problem<br>What are the patient's feelings concerning the complaint or problem.							
3. patient's expectations (i.e., goals, what help the patient had expected for each problem)							
4. Effects: how each problem affects the patient's life<br>What impact have significant others (partner, family, friends) or important living situations (work, hobbies, sports) had on all of the above questions?							
4 What has the patient himself done to manage the undesirable condition.							

EMOTIONS (item 9) (item 3 MAAS: score 0-6)<br>0 = not present /1 = poor /2 = unsatisfactory /3 = doubtful<br>4 = satisfactory/ 5 = good /6 = excellent							
Asking about/ exploring feelings reflecting feelings (including nature and intensity) sufficiently throughout the entire consultation							
General feeling/comments							
